# Three Classes of Plasmid (47–63 kb) Carry the Type B Neurotoxin Gene Cluster of Group II *Clostridium botulinum*

**DOI:** 10.1093/gbe/evu164

**Published:** 2014-07-30

**Authors:** Andrew T. Carter, John W. Austin, Kelly A. Weedmark, Cindi Corbett, Michael W. Peck

**Affiliations:** ^1^Gut Health and Food Safety, Institute of Food Research, Norwich Research Park, Norwich, United Kingdom; ^2^Bureau of Microbial Hazards, Health Products and Food Branch, Health Canada, Ottawa, Ontario, Canada; ^3^National Microbiology Laboratory, Public Health Agency of Canada, Winnipeg, Manitoba, Canada

**Keywords:** botulism, conjugation, plasmid toxin–antitoxin systems

## Abstract

Pulsed-field gel electrophoresis and DNA sequence analysis of 26 strains of Group II (nonproteolytic) *Clostridium botulinum* type B4 showed that 23 strains carried their neurotoxin gene cluster on a 47–63 kb plasmid (three strains lacked any hybridization signal for the neurotoxin gene, presumably having lost their plasmid). Unexpectedly, no neurotoxin genes were found on the chromosome. This apparent constraint on neurotoxin gene transfer to the chromosome stands in marked contrast to Group I *C. botulinum,* in which neurotoxin gene clusters are routinely found in both locations. The three main classes of type B4 plasmid identified in this study shared different regions of homology, but were unrelated to any Group I or Group III plasmid. An important evolutionary aspect firmly links plasmid class to geographical origin, with one class apparently dominant in marine environments, whereas a second class is dominant in European terrestrial environments. A third class of plasmid is a hybrid between the other two other classes, providing evidence for contact between these seemingly geographically separated populations. Mobility via conjugation has been previously demonstrated for the type B4 plasmid of strain Eklund 17B, and similar genes associated with conjugation are present in all type B4 plasmids now described. A plasmid toxin–antitoxin system *pemI* gene located close to the neurotoxin gene cluster and conserved in each type B4 plasmid class may be important in understanding the mechanism which regulates this unique and unexpected bias toward plasmid-borne neurotoxin genes in Group II *C. botulinum* type B4.

## Introduction

*Clostridium* botulinum is a highly pathogenic bacterium that forms the deadly botulinum neurotoxin. This is the most potent toxin known; ingestion of as little as 30–100 ng is potentially fatal to a human (Peck 2009). Eight distinct botulinum neurotoxin types (types A–H, although type H remains to be verified [Johnson 2014)], and more than 30 different neurotoxin subtypes (e.g., subtypes B1–B7) are recognized (Peck et al. 2011; Hill and Smith 2013; Peck 2014). The botulinum neurotoxins are 150 kDa proteins with zinc-endopeptidase activity that block acetylcholine transmission, leading to a potentially fatal floppy paralysis known as botulism. *Clostridium botulinum* is a heterogeneous species that comprises a complex of four distinct groups of bacteria that share the common property of forming the botulinum neurotoxin (Hatheway 1988; Johnson 2007; Peck 2014). Group I (proteolytic) *C. botulinum* and Group II (nonproteolytic) *C. botulinum* are responsible for most cases of foodborne botulism. Group II (nonproteolytic) *C. botulinum* is a psychrotroph with a minimum growth temperature of 2.5–3.0 °C, and strains form a single botulinum neurotoxin of type B, E, or F (Peck 2006; Peck et al. 2008; Lindström et al. 2009). All Group II *C. botulinum* type B strains exclusively form neurotoxin of subtype B4, with the other six type B neurotoxin subtypes formed by members of Group I *C. botulinum.* Several distinct subtypes of type E neurotoxin are formed by Group II *C. botulinum,* whereas all Group II *C. botulinum* type F strains uniquely form subtype F6 neurotoxin (Carter et al. 2013; Hill and Smith 2013; Stringer et al. 2013). Recently, it was shown that the type F6 strains also contain a remnant of a type B and a type E neurotoxin gene (Carter et al. 2013).

Outbreaks of foodborne botulism in Europe are frequently associated with type B neurotoxin (Hauschild 1993; Peck 2009; Mazuet et al. 2011). These often involve Group II *C. botulinum* type B and meat products (Sebald and Jouglard 1977; Lücke 1984; Hauschild 1989; Lund and Peck 2013). In 1895, van Ermengem isolated the first strain of Group II *C. botulinum* following an outbreak of foodborne botulism in Belgium associated with salted ham (van Ermengem 1979). It is probable that this was a strain of Group II *C. botulinum* type B. Two outbreaks in Iceland in 1981 and 1983, at least one associated with blood sausages, were also caused by Group II *C. botulinum* type B (Maslanka S, Personal communication). More recent outbreaks of foodborne botulism associated with Group II *C. botulinum* type B have involved a jar of home-preserved pork brought in to the United Kingdom from Poland (McLauchlin et al. 2006) and home-prepared ham in France (Mazuet et al. 2014). Outbreaks of foodborne botulism associated with Group II *C. botulinum* type B have also involved fish in North America, such as those involving salmon eggs (Dolman et al. 1960), salted salmon (CDC 1979), trout (Hauschild 1985), and dried salted whole whitefish (kapchunka) (CSDHS 1981; CDC 1985).

The botulinum neurotoxin gene is located within the neurotoxin gene cluster, together with other genes that encode accessory proteins. There are two distinct neurotoxin gene clusters. The *ha* (haemagglutinin) type, possesses an operon of three *ha* genes, which is transcribed in the opposite direction to a second operon comprising the nontoxin nonhaemagglutinin gene and the neurotoxin gene. Separating these two divergent operons is a small gene encoding the sigma factor, BotR, which is responsible for activating expression of each operon; this gene is transcribed in the same orientation as the neurotoxin-encoding operon (Peck et al. 2011; Hill and Smith 2013). All type B neurotoxin genes are located in an *ha* neurotoxin gene cluster (Carter et al. 2009; Peck 2009; Hill and Smith 2013; Stringer et al. 2013). The *orfX* type of neurotoxin gene cluster replaces the *ha* operon with three genes of unknown function, named *orfX*, which are separated from the *botR* gene by the remains of a transposon insertion sequence (IS) element. In this case, *botR* is transcribed in the opposite orientation to the neurotoxin-encoding operon, and is separated from this by an extra gene, also of unknown function and named after the size of its gene product, p47 (Hill et al. 2009). Neurotoxin gene clusters are often flanked by the remains of mobile genetic elements, and this and other genetic evidence suggests that they have been acquired by horizontal gene transfer (Sebaihia et al. 2007; Carter et al. 2009). *Clostridium botulinum* neurotoxin gene clusters may be located on the bacterial chromosome, on a plasmid or in a bacteriophage genome maintained in the cell as a plasmid (Peck et al. 2011). For Group II *C. botulinum,* the type B neurotoxin gene of the two strains examined to date was located on a plasmid (Franciosa et al. 2009), whereas the type E neurotoxin gene was found on the chromosome or a plasmid (Zhang et al. 2013), and the type F neurotoxin gene was chromosomally located (Carter et al. 2013).

Of the *C. botulinum* plasmids that bear a neurotoxin gene cluster, those of Group I *C. botulinum* not only share blocks of synteny, but also similar sites of insertion of the neurotoxin gene cluster (Hill et al. 2009; Dover et al. 2013). No synteny exists between plasmids of Group I *C. botulinum* and Group II *C. botulinum,* although only the plasmid of Group II *C. botulinum* type B strain Eklund 17B has been studied (Hill et al. 2009; Stringer et al. 2013); however, it has been shown that examples of both Group I *C. botulinum* neurotoxin gene cluster-bearing plasmids and pCLL of Group II *C. botulinum* type B strain Eklund 17B are all mobilisable by conjugation (Marshall et al. 2010). Three strains of Group II *C. botulinum* type E1 have been identified as bearing the neurotoxin gene on a plasmid (Zhang et al. 2013), but no sequence data for these plasmids are available. The only significant DNA homology (i.e., >70%) shared between the plasmid of Eklund 17B and any of the five plasmids of Group III type C/D strain BKT015925 (Skarin et al. 2011) is confined to the neurotoxin gene cluster (78–83% bp identity) located on the 203-kb bacteriophage plasmid p1BKT015925 (Carter A, Unpublished data).

In a recent comparative whole-genome study of Group II *C. botulinum* types B, E, and F, using a DNA microarray (Stringer et al. 2013) we noted that some Group II *C. botulinum* type B strains gave hybridization patterns which lacked any signal for plasmid genes other than those of the type B4 neurotoxin gene cluster itself. This was entirely unexpected and suggested either that this gene cluster was carried by the chromosome or that it was on a plasmid that bore little sequence homology to the only published example, that of strain Eklund 17B (Stringer et al. 2013). Building on this microarray study, we have established for a number of representative strains of Group II *C. botulinum* type B that all contain the type B4 neurotoxin gene, which is located on a plasmid. We have used genome sequencing to characterize these neurotoxin gene cluster-bearing plasmids and have explored the evolutionary relationship between the plasmids and the environment from which their bacterial hosts were isolated.

## Materials and Methods

### Bacterial Strains and Growth

Strains of Group II (nonproteolytic) *C. botulinum* type B used in this study are listed in table 1. Strains Kapchunka B3, Kapchunka B8, DB2, and Eklund 2B were maintained frozen at −80 °C on Microbank beads (Pro-Lab Diagnostics, Richmond Hill, ON), and before use were checked for purity by growth on McClung Toabe agar plates supplemented with egg yolk, incubated under aerobic and anaerobic atmospheres (Daifas et al. 2004). All other strains were kept as spore stocks in Robertson’s cooked meat broth (RCMB) at 4 °C, and prior to use were checked for purity by growth on PYGS (peptone, yeast extract, glucose, starch) and Reinforced Clostridial Medium with 5% (w/v) skim milk agar plates incubated under aerobic and anaerobic conditions (Stringer et al. 2009). For pulsed-field gel electrophoresis (PFGE) DNA preparations, 100 µl-spores from an RCMB stock were inoculated into 10-ml TPYG (tryptone, peptone, yeast extract, glucose) broth and incubated at 30 °C overnight. The next day, 5 µl of the overnight culture was inoculated into 10-ml TPYG broth and incubated at 30 °C until the OD_600_ reached 1.8–2.0. Cells were then harvested for PFGE.

**Table 1:** Details of Group II *Clostridium botulinum* type B4 Strains Analysed

Strain Name	Isolation Details			Linked to Botulism Outbreak	Received from^a^
	Source	Location	Date		
2129B^b^	Unknown	France	1950s?	?	NFPA
Eklund 17B	Pacific sediments	USA	1965	No	NCIMB
Eklund 2B	Pacific sediments	USA	1965	No	UR/FDA
DB2^c^	Pacific sediments	USA	1968	No	FDA
Colworth BL151	Haddock	Norway	1960s?	No	UR
Hobbs FT50	Herring	UK	1960s?	No	UR
ATCC 17844	Unknown	USA?	<1970s?	?	UH
CDC 706	Fermented salmon brine from Alaska	USA	1976	Yes	CDC
Kapchunka B2	Dried salted whole whitefish [Kapchunka]	USA	1981	Yes	NFPA
Kapchunka B3^c^	Dried salted whole whitefish [Kapchunka]	USA	1981	Yes	FDA
Kapchunka B5	Dried salted whole whitefish [Kapchunka]	USA	1981	Yes	NFPA
Kapchunka B8^c^	Dried salted whole whitefish [Kapchunka]	USA	1981	Yes	FDA
Kapchunka B9	Dried salted whole whitefish [Kapchunka]	USA	1981	Yes	NFPA
CDC 3875	Human stool from botulism case	Iceland	1981	Yes	CDC
CDC 3897	Human stool from botulism case	Iceland	1981	Yes	CDC
CDC 4627 U-1	Whey used to pickle/store blood sausage	Iceland	1983	Yes	CDC
CDC 5900	Human stool from botulism case (ham?)	Italy	1986	Yes	CDC
CDC 5900 A-T-A3	Human stool from botulism case (ham?)	Italy	1986	Yes	CDC
CB-S-30E	Fishpond sediment	Finland	1996	No	UH
IFR 05/020	Scallops (from Canada)	UK	2005	No	IFR
IFR 05/024	Scallops (from Canada)	UK	2005	No	IFR
IFR 05/025	Dried egg pasta (Fettucine from Italy)	UK	2005	No	IFR
IFR 05/029	Dried egg pasta (Trucioli from Italy)	UK	2005	No	IFR
IFR 05/035	Dried egg pasta (Trucioli from Italy)	UK	2005	No	IFR
H1 5088 0797	Jar of home cooked pork (from Poland)	UK	2005	Yes	PHE
IFR 12/036	Honeycomb honey (from Poland)	UK	2012	No	IFR

^a^Originally from: CDC, C. Hatheway (Centers for Disease Control, USA); IFR, isolated at IFR; NCIMB, National Collection of Industrial, Food and Marine Bacteria, UK; NFPA, V. Scott (National Food Processors Association, USA); UH, M. Lindström (University of Helsinki, Finland); UR, J. Crowther (Unilever Research, UK). PHE (K. Grant, Public Health England, London, UK); FDA, Haim Solomon (U.S. Food and Drug Administration, USA). Kapchunka B2, B3, B5, B8, and B9 are different strains from the same outbreak (that might be identical). CDC 5900 A-T-A3 and CDC 5900 are different strains from the same stool sample (that might be identical). CDC 3875 and CDC 3897 were from the stool of different individuals involved in the same botulism outbreak.

^b^A synonym for this strain is Prevot 59, which was previously reported to carry a type B2 neurotoxin gene (Hill et al. 2007). We have sequenced the neurotoxin gene of our version of this strain and found it to be type B4 (Stringer et al. 2013).

^c^Strains not screened by PFGE before genome sequence analysis.

### Pulsed-Field Gel Electrophoresis

To inactivate nucleases, 1 ml formalin (37% formaldehyde solution) was added to each 10 ml cell culture. After mixing, cells were chilled at 4 °C, 30 min, and then pelleted by centrifugation for 10 min at 4,200 × g, 4 °C. Cell pellets were washed twice in 10 ml ice-cold 0.85% NaCl, and then resuspended in 0.5 ml Resuspension Buffer (10 mM Tris–HCl, pH 8.0, 1 M NaCl, 100 mM ethylenediaminetetraacetic acid [EDTA]). After incubation at 50 °C for 10 min, cells were mixed with an equal volume of 2% Bio-Rad Chromosomal Grade Agarose (162-0135) made up in Resuspension Buffer and cooled to 50 °C. The bacterial cell/agarose mixture was loaded into Bio-Rad plug moulds (CHEF Mapper XA system 50-well Plug Mould, 170-3713). Moulds were chilled at 4 °C for 15 min, and then plugs were removed and incubated in four volumes of Lysis Buffer (10 mM Tris–HCl, pH 8.0, 1M NaCl, 100 mM EDTA, 0.5% Brij 58, 0.2% sodium deoxycholate, 0.5% n-lauryl sarcosine, 100 µg/ml RNase A, 5 mg/ml lysozyme, 40 U/ml mutanolysin) at 37 °C, 12–18 h. Lysis buffer was removed and plugs were rinsed with 50 mM EDTA solution to remove enzymes. Two volumes proteinase K (PK) buffer (10 mM Tris–HCl, pH 8.0, 0.5 M EDTA, 1% n-lauryl sarcosine, 1 mg/ml Proteinase K [Qiagen 19131]) was added, and plugs incubated at 50 °C, 48 h, with gentle rotary agitation. Plugs were rinsed with Tris EDTA (TE) buffer (10 mM Tris–HCl, pH 8.0, 1 mM EDTA), then incubated at 37 °C, 2 h with gentle shaking in TE buffer containing 1 mM phenylmethylsulphonyl fluoride (PMSF, Fluka 93482, 0.1 M in ethanol) to inactivate Proteinase K. Finally, plugs were washed 3 × 2 h in ten volumes TE buffer to remove PMSF. PFGE was carried out in a 1% agarose gel (PFGE grade agarose, Bio-Rad 162-0137) using a Bio-Rad CHEF-DR II system, in 0.5 × TBE (Tris Borate EDTA, manufacturer’s recipe) with the following parameters: 6 V/cm, initial switch 1 s, final switch 26 s, run time 20 h at 14 °C (Hielm et al. 1998).

### Southern Analysis

PFGE gels were stained with ethidium bromide, 0.5 µg/ml in water, 30 min and then photographed under shortwave UV light. DNA was partly depurinated by gentle agitation in 0.25 M HCl, 10 min, and then denatured by soaking gels in 1.5 M NaCl, 0.5 M NaOH, 30 min, before neutralization in 1 M Tris–HCl (pH 8), 1.5 M NaCl, 30 min, all at room temperature. Gels were blotted overnight onto nylon membrane using a Turbo-Blotter capillary system (Schleicher and Schuell) and 20× SSC transfer buffer. Southern blots were rinsed, 1 min in 2× SSC, and then blotted dry on Whatman 3 MM paper. DNA was fixed to the membrane by UV cross-linking (auto setting, Stratalinker 2400, Stratagene). Prehybridization, hybridization (42 °C), and DNA probe labeling were performed using an Amersham ECL Direct Nucleic Acid Labeling and Detection System (GE Healthcare). Probe DNA to detect Group II *C. botulinum* type B4 genes was prepared by polymerase chain reaction (PCR) using Eklund 17B genomic DNA (100 ng) as template, amplifying two regions of the Eklund 17B neurotoxin gene. Primer pairs were: 5′-GGGGAGATATTATAAAGCTTTTAAAATCA-3′ and 5′-TTAGGAATAAACTGCCAATTACATCC-3′, which give a 3,764-bp product, and: 5′-GGGGAGATATTATAAAGCTTTTAAAATCA-3′ and 5′-GGAAATGTCTGAGAGTATAAATATTGAAA-3′, which give a 1,550-bp product. An aliquot (200 ng) of each PCR product was labeled for use as probe in each Southern hybridization. Probe DNA to detect chromosomally located 16S ribosomal RNA genes was amplified from Group II *C. botulinum* type B strain Colworth BL151 (100 ng) using PCR primer pair 5′-GATACCCTGGTAGTCCACGC-3′ and 5′-GCACACGGTTTCAGGTTCTA-3′, which produce a 1.5-kb PCR product.

## DNA Sequencing

For strains CDC 3875, IFR 05/025, Colworth BL151, Kapchunka B2, CDC 5900, and CDC 3897, genomic DNA was extracted from 10-ml TPYG broth cultures using standard Gram positive cell lysis procedures followed by phenol–chloroform purification (Sebaihia et al. 2007). For strains Kapchunka B3, Kapchunka B8, DB2, and Eklund 2B, genomic DNA was prepared using the Qiagen DNEasy Blood & Tissue kit (Qiagen), from a single colony grown on TPYG agar, and libraries were prepared using the Illumina Nextera DNA Sample Prep Kit (Illumina) according to manufacturer instructions. All genome sequencing was carried out using the Illumina MiSeq platform. Strains CDC 3875 and IFR 05/025 were sequenced at The Genome Analysis Centre, Norwich, UK, with reads assembled into contigs using the short-read assembler ABySS 1.3.4 (Simpson et al. 2009). Strains Colworth BL151, Kapchunka B2, CDC 5900, and CDC 3897 were sequenced by Eurofins Genomics, and the reads assembled using Velvet 1.2.10 (Zerbino and Birney 2008). Strains Kapchunka B3, Kapchunka B8, DB2, and Eklund 2B were sequenced at Health Canada, with reads assembled using the De Novo Assembly programme of CLC Genomics Workbench 6.5.1. Our sequencing and finishing of the chromosome and plasmid (pCB17B) of strain Eklund 17B (NRP) has been described previously (Stringer et al. 2013).

### Assembly and Annotation of Plasmid Contigs

Contigs which included the type B4 neurotoxin gene were identified by searching with sequence from the B4 gene of Eklund 17B (accession number FR745876); this was done by loading the contig file in FASTA format into Microsoft Word 2010 and using short sections of the type B4 gene-coding sequence (including the reverse/complement) to perform a word search. If no overlapping contig was available, the raw data fastq reads were used to extend both ends of these contigs until a neighboring contig was found. This was done by loading the fastq reads file into Microsoft Excel 2010 and word-searching for short lengths of DNA sequence from the end of the contig. Reads containing this sequence were assembled in Vector NTI Advance 11 using the default settings of the programme ContigExpress, to form a new contig which could then be added to the original one. This process was repeated until, in each case, the opposite end of the contig was discovered, indicating that a circle could be formed. All putative contig junctions were confirmed by PCR across the junction, followed by capillary sequencing of the resulting PCR product. Contig junction regions were also compared with sequences of closely related or identical plasmids for further confirmation that this approach was robust. Sequence comparisons were performed using Artemis Comparison Tool (ACT) using parameters that showed blocks of nucleotide identity of >90% (Carver et al. 2005). Coding sequences (CDSs) which spanned a contig junction were checked by BLAST analysis for any open-reading frame (ORF) disruption, which might have indicated incorrect assembly. Initial ORF identification for each plasmid was carried out, and phylogenetic trees were constructed using Vector NTI Advance 11 software. Annotation was carried out using Glimmer 3.02 from the x-BASE bacterial genome annotation service (Delcher et al. 2007), the RAST server (Aziz et al. 2008), together with manual BLASTp (Altschul et al. 1990) and DELTA BLAST (Boratyn et al. 2012) searches (NCBI) using peptide sequence derived from predicted coding sequences. Plasmid TA genes were assigned using the web-based search tool RASTA-Bacteria (Sevin and Barloy-Hubler 2007). Estimates of plasmid copy number were performed using the De Novo Sequencing, “Map Reads to Contigs” tool of CLC Genomics Workbench 6.5.1. The number of Illumina fastq reads passing the default QC (1.6–2.4 million reads per genome) which mapped onto each finished plasmid sequence was compared with the number which mapped to chromosomal DNA. The ratio of these two results, normalized to map units per base pair, gave an approximate value for plasmid copy number at the time of preparation of genomic DNA.

## Results

### Group II *C. botulinum* Type B4 Neurotoxin Genes Are Extrachromosomally Located

**Fig. 1.—:**
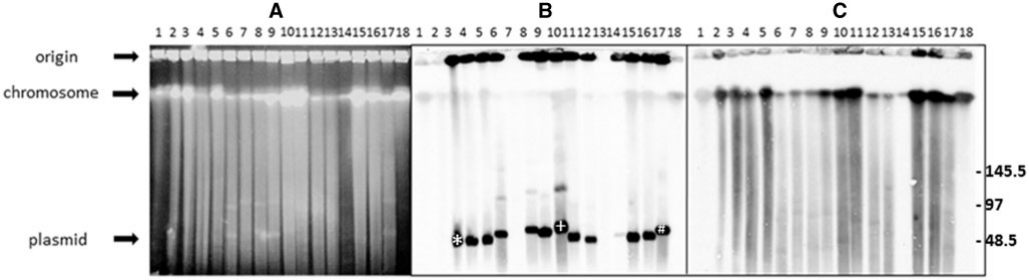
PFGE analysis shows that the type B neurotoxin gene is not located on the chromosome. (*A*) Stained PFGE gel in which intact chromosomal DNA is separated from extrachromosomal DNA (faint bands at bottom). Southern blot of same gel, (*B*) probed for type B neurotoxin gene; (*C*) reprobed for chromosomally located rRNA genes. Lanes 1, NCTC 11199; 2, Hobbs FT50; 3, Eklund 17B; 4, Eklund 2B; 5, Colworth BL151; 6, CDC 3875; 7, CDC 4627 U-1; 8, CDC 5900 A-T-A3; 9, 2129B; 10, CDC 5900; 11, Kapchunka B2; 12, Kapchunka B5; 13, CB-S-30E; 14, ATCC 17844; 15, IFR 05/020; 16, IFR 05/024; 17, IFR 05/025; 18, MDa10. Lanes 1 and 18 contain DNA from strains of Group I *Clostridium botulinum* type A (*B*). Bands in panel *B* marked with white symbols are of known size as determined from their DNA sequence: (_*_), 47.7 kb; (+), 63 kb; (#), 60.6 kb. Horizontal dashes at right of panel *C* indicate approximate positions and size (kb) of whole-phage lambda size markers.

The genomic DNA of 23 Group II *C. botulinum* type B4 strains (all those in table 1, except for Kapchunka B3, Kapchunka B8, and DB2) was screened by PFGE using parameters designed to separate intact chromosomal DNA from any extrachromosomal DNA. The ethidium bromide-stained PFGE gel (fig. 1*A*) was then subjected to Southern-blot analysis (fig. 1*B*). Probes for the type B4 neurotoxin gene showed that all strains carried this exclusively on an extrachromosomal element. By comparison with a PFGE phage lambda size ladder and with signal from the previously sequenced plasmid of strain Eklund 17B on the Southern blots, these extrachromosomal elements were estimated to range between approximately 47 and 63 kb (fig. 1*B*). Importantly, no examples were found which possessed a chromosomal location for this gene. To confirm that intact chromosomal DNA had been successfully transferred to each blot, these were rehybridized with a probe for 16S rDNA, which in all *C. botulinum* genomes so far sequenced is chromosomally located (fig. 1*C*). Three *C. botulinum* strains; Hobbs FT50, CDC 4627 U-1, and CB-S-30E generated no signal when their DNA was probed for the neurotoxin gene; this is attributed to loss of the presumed extrachromosomal element which bears it. This result confirmed observations from our previous microarray analysis (Stringer et al. 2013), where it was noted that some strains designated as type B gave no microarray signal with probes for the neurotoxin gene cluster.

### Genome Sequencing Confirms that Group II *C. botulinum* Type B4 Neurotoxin Genes Are Carried by Plasmids

Six strains that had tested positive for the type B4 neurotoxin gene by PFGE/Southern analysis were sequenced. These were chosen to represent not only different-sized extrachromosomal elements, as estimated by PFGE, but also different genetic backgrounds, as determined by microarray analysis (Stringer et al. 2013). In addition, the genomes of four further *C. botulinum* type B4 strains were sequenced. These genome sequences were added to that of our published strain Eklund 17B (NRP) (Stringer et al. 2013), making a total of 11 genomes available for comparison. All sequencing was performed using the Illumina MiSeq platform, and the paired-end reads generated by this technology were assembled de novo into contigs. Contigs containing the type B4 neurotoxin gene cluster were identified and used as the starting point for construction of the predicted extrachromosomal element. In each case this element formed circular DNA, which annotation (see below) showed to be a plasmid. The sizes of these plasmids determined by sequencing agreed closely with the estimates obtained from PFGE/Southern analysis (fig. 1). Assuming that each plasmid was recovered at a similar efficiency to that of genomic DNA during the extraction procedure, the average fold coverage of paired-end reads to either chromosomal (chosen at random) or plasmid contigs suggested that each plasmid copy number is of the low to medium range, approximately 2–5 copies per cell (data not shown).

### Plasmids Carrying the Type B4 Neurotoxin Cluster Belong to One of Three Main Classes

**Fig. 2.—:**
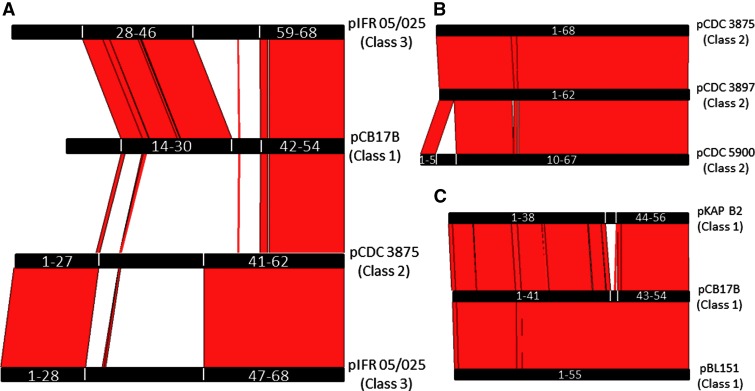
ACT modified screen shots showing regions of sequence homology (>90% nt sequence identity) shared between plasmids representing the three classes found in Group II *Clostridium botulinum* type B4. All classes share the neurotoxin gene cluster, which comprises approximately one-third of each plasmid and is located at the right hand end of each sequence (for example in pCB17B, genes 47–52). (*A*) Plasmid pIFR 05/025 is represented twice, to demonstrate the separate blocks of sequence that it shares with members of the two other plasmid classes. Plasmid pCB17B is carried by Eklund 17B (NRP). (*B* and *C*) Members of the two most common classes of Group II *C. botulinum* type B4 plasmids share much greater homology, with differences due to insertion/deletion of a small number of genes. The four extra genes carried by pCDC 5900 (when compared with pCDC 3897) are probably all phage-derived (three integrases and a transposase).

**Fig. 3.—:**
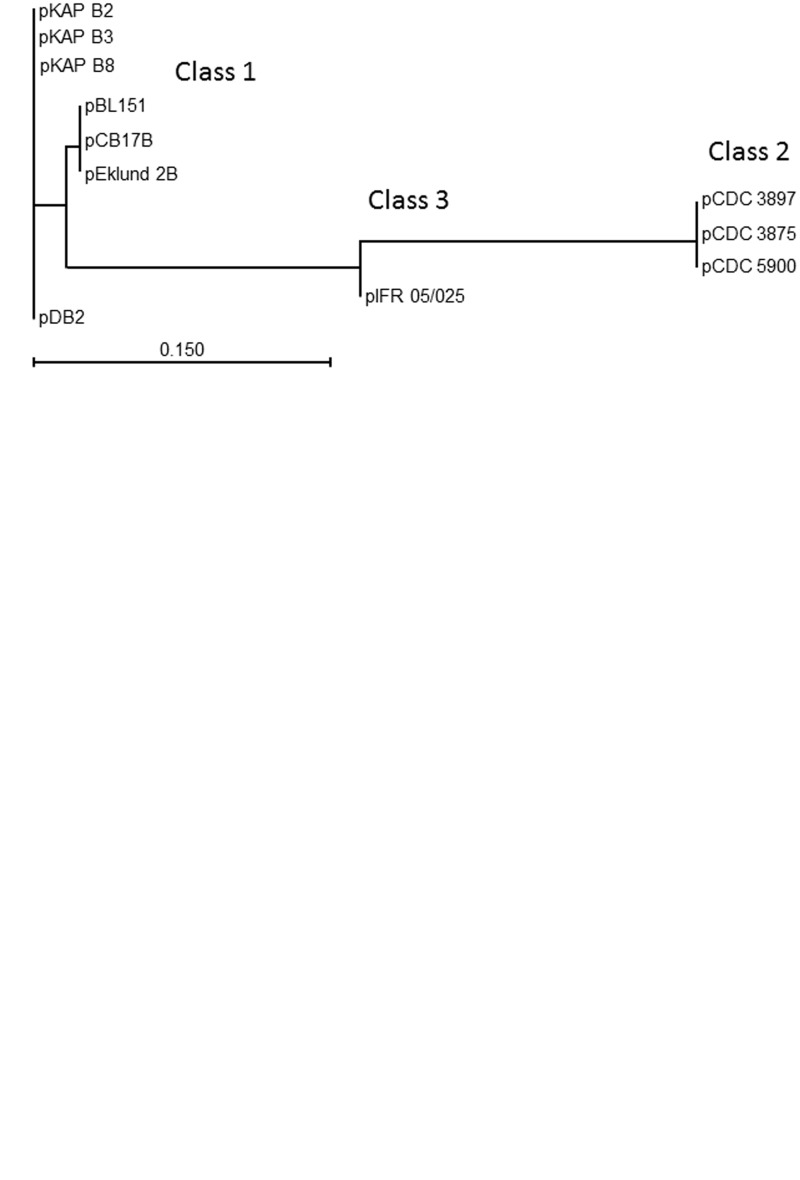
(*A*) Unrooted neighbour-joining (Jukes–Cantor) tree showing the three main plasmid classes found in Group II *Clostridium botulinum* type B4. Plasmid pIFR 05/025 shares approximately 71% identity with the Eklund 17B class and 67% identity with the pCDC 3875 class. The differences between members of classes 2 and 3 due to insertions/deletions, apparent in figure 2 are too small to be seen on this scale. The scale bar indicates branch length. Plasmids pKAP B2, pKAP B3, and pKAP B8 are 48,298 bp and are identical; pDB2 is 48,290 bp and shares 99.9% identity with the pKAP B series. Plasmids pBL151 and pEklund 2B are 46,642 bp and identical; pCB17B is 47,689 bp and differs from these by 3.7%. Plasmids pCDC 3897 and pCDC 3875 are 58,175 bp and identical; pCDC 5900 is 62,986 bp and differs from the other plasmids of class 2 by 7.6%. (*B*) Unrooted neighbour-joining (Jukes–Cantor) neurotoxin gene cluster tree. The entire neurotoxin gene cluster was included in the alignment, ranging from the 3′-end of the ha70 gene to the 3′-end of the boNT/B4 gene (11675 bp in each case). Number of SNPs defining each branch length is indicated. Note that IFR 05/025 now clusters with the CDC 3875 class. Plasmid class for each neurotoxin gene cluster is in parentheses. The scale bar indicates branch length. (*C*) Unrooted neighbour-joining (Jukes–Cantor) type B4 neurotoxin gene tree. DNA sequences not determined in this study were obtained from GenBank (NCBI). Number of SNPs defining each branch length is indicated. The sequence with accession number X71343 was published as being that of Eklund 17B (Hutson et al. 1994); however, it differs from the two other published versions (which are identical) by 21 bases. Attempts to locate the organism used in this study were unsuccessful. The scale bar indicates branch length. SNPs, single nucleotide polymorphisms.

Complete plasmid sequences were compared using ACT (fig. 2). This revealed two different levels of relatedness between the plasmids. Evidence of relatively minor variation was small insertion/deletion events involving up to four CDSs, plus more scattered sequence differences, mostly single nucleotide polymorphisms (data not shown), presumably indicative of genetic drift. The parameters of the ACT analysis depicted in figure 2 are set so that sequence identity of approximately 90% or more appears as a solid red block connecting each pairwise comparison, so at the degree of magnification used in figure 2, the latter form of genetic variation is masked. Allowing for up to 10% sequence differences, the plasmids could be organized into three main classes; class 1, represented by pCB17B of Eklund 17B comprised seven members, each of approximately 48 kb; class 2, represented by pCDC 3875 comprised three members of sizes between approximately 58–63 kb; class 3 is represented by its only member, pIFR 05/025 (60 kb), which appeared to be a hybrid version of the two other classes. Allowing for the genetic drift already discussed, the regions of similarity between each plasmid class are quite marked, being defined by large blocks of sequence rather than by regions of intermittent homology (fig. 2*A*). Figures 2*B* and *C* demonstrate the high degree of relatedness between members within a single class, a fact which facilitated identification of the inserted/deleted CDSs. DNA sequences were used to generate phylogenetic trees; of the complete plasmid (fig. 3*A*), the neurotoxin gene cluster (fig. 3*B*) and, together with those also available in GenBank, the type B4 neurotoxin gene (fig. 3*C*). Three distinct groups of neurotoxin gene cluster and neurotoxin genes were identified (figs. 3*B* and *C*). As expected for Group II *C. botulinum* type B, all neurotoxin genes were of subtype B4; their DNA sequence variation fell within the range for subtype B4 (<48 nt) observed in a previous study of *C. botulinum* neurotoxin gene diversity (Hill et al. 2007).

### Gene Annotation Suggests that Type B4 Plasmids Are Conjugative

**Fig. 4.—:**
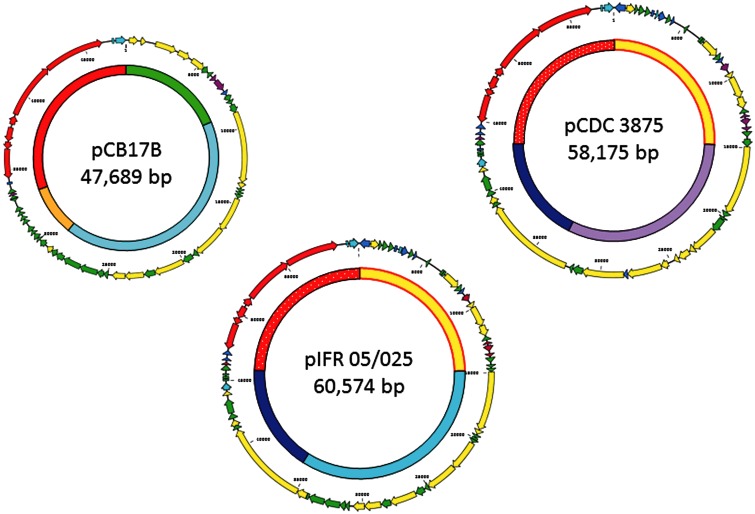
Coding regions shared by plasmids of Group II *Clostridium botulinum* type B4. Maps depict a typical member of each plasmid class described in the present work. Outer circles; CDSs colored according to their predicted gene function (see GenBank files for full annotation). Red; Type B4 neurotoxin gene cluster. Yellow; plasmid replication/conjugation. Green; gene product of unknown function. Blue; known gene product not exclusively associated with a plasmid. Light blue; bacteriophage gene/IS element. Purple; TA system. Inner circles; color-coded map delineating regions of high (>90%) DNA homology shared by different plasmid classes. For example, a 15-kb region shared by pCDC 3875 and by pIFR 05/025 (yellow with red border); a 20-kb region shared by pCB17B and by pIFR 05/025 (light blue). The 15-kb region colored red, shared by all three classes, contains the neurotoxin gene cluster plus several 5′-flanking ORFs; this region in pIFR 05/025 and pCDC 3875 has been stippled to show their closer homology. It can be seen that three regions are unique; a 9- and a 4-kb region in pCB17B (green and orange, respectively), and an 18.5-kb region in pCDC 3875 (lilac). An 8-kb region of pCB17B (genes P007–P016) also shares homology with the other plasmids, but at only 58% this is not shown.

The DNA sequence of each plasmid was used to predict ORFs. These ORFs were annotated by several different approaches to gain a consensus prediction of their gene products. The resulting maps of three plasmids, each one a typical representative of its class, have been color coded to visualize the distribution of gene types discovered by this process (fig. 4). The colored circles located within each plasmid map represent the same DNA identity data originally generated by ACT comparison (fig. 2), but here are color-coded to highlight regions that are shared by each plasmid class. When juxtaposed next to the plasmid gene maps these syntenic regions of shared genes become more obvious (fig. 4).

A large proportion (37–43% of each plasmid [fig. 4]) of plasmid DNA sequence not associated with the neurotoxin gene cluster itself is devoted to genes whose products are predicted to be involved in plasmid replication and/or mobilization by conjugation. The more recognizable candidates for conjugative processes are listed in table 2. PspC-like proteins can be involved in cell wall binding, so may facilitate binding of the conjugation pilus. Other candidates for cell wall binding are the NlpC family protein and the transglutaminase. The relaxase initiates conjugation by binding to the origin of transfer (*oriT*), a process which is facilitated by MobC. The relaxase/DNA complex binds to type IV coupling proteins, which are supplied by TraG/VirD gene products, possibly also the N-terminal transmembrane domain, C-terminal repeat protein. The type IV secretion system that mediates transfer of DNA via the pilus or conjugation tube is a complex of several proteins, candidates for which include VirB proteins, PrgI, and the lytic transglycosylase. Products of the large helicase genes may act in concert with the single-stranded DNA-binding proteins to perform an equivalent function to that of TraI, which is bifunctional, possessing relaxase activity and catalysing a processive 5′- to 3-′ helicase reaction that provides the motive force for strand transfer. The presence of these genes suggests that each plasmid may have a functional conjugational system.

**Table 2:** Plasmid-Encoded Predicted Gene Products Likely to be Involved in Conjugation

pCB17B (Class 1)	pCDC 3875 (Class 2)	pIFR 05/025 (Class 3)
P15: PspC-like protein	P18: MobC relaxase	P18: MobC relaxase
P20: TraG/VirD	P19: relaxase	P19: relaxase
P21: N-terminal transmembrane domain, C-terminal repeat protein	P20: transglutaminase	P20: transglutaminase
P23: VirB4-like protein	P27: PspC-like protein	P28,29: PspC-like protein
P26: NlpC family protein	P31: TraG/VirD4	P33: TraG/VirD
	P32: VirB2	P34: N-terminal transmembrane domain, C-terminal repeat protein
	P33: VirB6	P37: VirB
	P34: PrgI	P39: NlpC family protein
	P35,36: TraE/VirB4	P46: HicB domain-containing protein
	P38: lytic transglycosylase	P47: helicase
	P41: helicase	P49,52: single stranded binding protein
	P43,46: single stranded binding protein	

Note.—Plasmid classes 1–3 and their membership are defined in figure 3 and its legend. Gene number, (PX), refers to numbering in the respective GenBank file for each plasmid.

### Toxin–Antitoxin Genes

Toxin-antitoxin (TA) systems are often found in prokaryotic plasmids as mechanisms to ensure stable inheritance. The annotation procedure suggested the presence of several different plasmid-based Type 2 TA systems. One TA gene cluster, only shared by plasmid class 2 and 3, contains three antitoxin genes; in the class 3 plasmid pIFR 05/025, top BLASTp hits for gene products of IFR 05/025_P22–P24 are the antitoxin component of the HicB TA system superfamily, followed by two antitoxin components of the Xre TA system family, respectively. Another TA gene also carried by the same two plasmid classes is of the zeta toxin type; for example in p05/025, the predicted gene product of IFR 05/025_P17 has 22% identity to the PezT zeta toxin of *Streptococcus vestibularis*. However, no cognate antitoxin partner was identified, suggesting that this is an orphan TA system component that is nonfunctional. Only one TA gene, *pemI* antitoxin of the PemI–PemK family, was shared by all 11 plasmids in this study (locus tag examples; class 1, CB17B_P044; class 2, CDC 3875_P52; class 3, 05/025_P58); this gene lies within the 15-kb region also containing the neurotoxin gene cluster that is conserved throughout the three type B4 plasmid classes (region colored red in fig. 4). No gene encoding a toxin cognate for *pemI* was apparent, again suggesting this is an orphan gene. The chromosomal DNA of Eklund 17B contains a gene annotated as a homologue of the toxin gene *pemK* (CB17B3278 in Eklund 17B (NRP), CLL_A3362 in B strain Eklund 17B). The antitoxin component of this system is annotated as such in the latter strain (CLL_A3363), but as a conserved hypothetical protein in the former (CB17B3277). Analysis of probe signal data generated by our comparative genomic microarray study (Stringer et al. 2013) showed that homologues of both of these genes are present in the genome of all 14 Group II *C. botulinum* type B4 strains examined (Unpublished data). Because the chromosomal *pemI*–*pemK* module seems to be intact, this raises an interesting question regarding the role that might be played by the plasmid-encoded orphan antitoxin PemI protein.

## Discussion

PFGE and Southern-blot analysis, combined with genome sequencing produced the unexpected result that all strains of Group II *C. botulinum* that possessed the type B4 neurotoxin gene carried it on a plasmid. None bore it on the chromosome, in marked contrast to Group I *C. botulinum*, which has several members with chromosomal neurotoxin cluster(s), plasmid neurotoxin cluster(s), or both (Hill et al. 2009; Dover et al. 2013). A search for plasmid-borne type B neurotoxin genes was carried out by Franciosa et al. (2009); however, the majority of strains in this study were of Group I *C. botulinum*. Only two strains (CDC 4848 and CDC 706) were of Group II *C. botulinum.* Both of these strains carried their neurotoxin gene on a plasmid. Unlike Group I *C. botulinum* neurotoxin gene cluster-bearing plasmids, which range from approximately 149 kb (pCLD of type B1 strain Okra) to 270 kb (pCLJ1 of Ba4 strain CDC 657), those of Group II *C. botulinum* type B4 clustered within a much smaller size range, between 47 and 63 kb. This also contrasts with the neurotoxin gene cluster-bearing plasmids recently identified in three Group II *C. botulinum* type E1 strains, which were estimated to be about 146 kb (Zhang et al. 2013).

DNA sequence comparison of the 11 plasmids showed that there were three classes, with the two most commonly found classes sharing only the neurotoxin gene cluster and a few flanking genes, these making up about a third of the total plasmid genetic content. Interestingly, the plasmid of strain IFR 05/025 represented a hybrid class which shared two-thirds of its DNA sequence with each of the other classes; although not the same two thirds in each case. In this respect, the Group II *C. botulinum* type B4 plasmids are similar to plasmids of Group I *C. botulinum,* which have been shown to share blocks of sequence but to also possess blocks that are unique (Hill et al. 2009). This implies that at some stage during the evolution of Group II *C. botulinum,* cells have contained more than one plasmid, and that these have undergone genetic recombination to form new plasmid varieties. The fact that 11 different strains of Group II *C. botulinum* type B4 only effectively carry three classes of plasmid between them, and that these also exhibit a limited size range, implies that plasmid recombination is a relatively rare event. If it were more common, one might expect to find both a greater size range and variety of genetic content. There is precedent for clostridial cells hosting multiple types of plasmid; several examples of Group I *C. botulinum* and Group III *C. botulinum* do so; the greatest number so far reported is in one member of Group III *C. botulinum* which has five (Skarin et al. 2011). To date, only one *C. botulinum* plasmid per genome bears a neurotoxin gene cluster, although there is an example of a single plasmid bearing more than one neurotoxin gene cluster; plasmid pCLJ1 of the Ba4 strain CDC 657 (Marshall et al. 2007; Smith et al. 2007). Other plasmids present in the same cell typically carry genes for fitness/survival factors such as bacteriocins (Sebaihia et al. 2007). An interesting and potentially important evolutionary link can be made between the three plasmid classes and the origin of the strains of Group II *C. botulinum* type B4 which carry them. All seven strains which carry a plasmid of class 1 originated from fish or marine sediments (primarily from the USA). All three strains which carry a plasmid of class 2 were from stool samples of human botulism cases involving meat products from Europe (Italy and Iceland). The interesting, apparently hybrid plasmid of class 3 was carried by a strain isolated in our laboratory from Italian egg pasta (Peck et al. 2010); this plasmid also shares its neurotoxin gene cluster and type B4 gene with the European class 2 group. This provides the first evidence for a link between geographic origin and a genetic subdivision within Group II *C. botulinum.*

In addition to defining the genetic relatedness of the type B4 neurotoxin-encoding plasmids, the phlyogenetic analysis showed that during plasmid evolution the neurotoxin gene cluster itself has acted as a separate genetic unit. In this respect, it is similar to findings for Group I *C. botulinum,* where a DNA microarray analysis showed that neurotoxin gene cluster type did not correlate with genetic background (Carter et al. 2009). The three type B4 neurotoxin gene subgroups identified in the present study are similar to those already described (Hill et al. 2007), and the fact that our study compares 17 type B4 genes in contrast to the five used for the previous analysis (Hill et al. 2007) suggests that any new type B4 genes described in the future may well fall within the same organization. Eight of the strains for which the type B4 neurotoxin gene sequence is available were also included in a whole-genome study using a DNA microarray (Stringer et al. 2013). Phylogenetic trees constructed using these microarray data showed that clades based on the whole genome correlated well with type of neurotoxin gene sequence, implying that the genetic linkage between the neurotoxin gene cluster and the chromosome is stronger than that with the plasmid. The three neurotoxin gene subgroups often reflect the origin of the strain. Four of six strains that cluster with the IFR 05/025 neurotoxin gene sequence were isolated following botulism outbreaks in Iceland, whereas IFR 05/025 itself was isolated from Italian egg pasta. Of the strains clustering with the KAP B2 neurotoxin gene sequence, five strains were isolated from botulism outbreaks associated with fish in North America, whereas one strain was from Pacific sediments.

All 11 plasmids studied here carried a set of genes which encode proteins necessary for conjugation; including relaxases, cell wall binding proteins, pilus formation proteins, cell wall lysis enzymes, and helicases. Plasmid class 1 possesses markedly fewer conjugation genes, particularly seeming to lack one for a relaxase. However, the “type” plasmid of class 1 is that of strain Eklund 17B, which has been experimentally proven to be transmissible in the laboratory by conjugation to a nontoxigenic strain of Group I *C. botulinum* (Marshall et al. 2010). Interestingly, the type B4 neurotoxin gene plasmids are not found outside of Group II *C. botulinum,* implying that in the environment the mobility of these plasmids is not broad host range. These type B4 plasmids are not found in strains of Group II *C. botulinum* type E or F (Stringer et al. 2013), possibly reflecting the restricted plasmid host range and/or host-encoded barriers to uptake of foreign DNA which appear to be very effective in Group II *C. botulinum.* Transmission by conjugation of a plasmid that lacks an obvious relaxase gene is uncommon, but there are at least four different examples known (Smillie et al. 2010), and the plasmids of class 1 may represent a fifth example. The presence of a *mobC* relaxase accessory gene in classes 2 and 3 ties in with the results of a wide-ranging study of conjugation systems, where it was found that *mobC* genes are specifically associated with midsized (20–60 kb) plasmids; the plasmids studied here fit within this size range (Smillie et al. 2010). Although the annotation process suggests that each of the plasmid classes seems to possess a different set of conjugation genes, the fact that all 11 plasmids are related, sharing large blocks of DNA sequence tends to suggest that the conjugation systems themselves will also be functionally closely related. A survey of conjugation systems (Smillie et al. 2010) noted that cointegration between plasmids is less damaging for long-term plasmid stability if the main components of the conjugation systems are from the same type.

The apparent evolutionary bias for strains of Group II *C. botulinum* type B4 to retain their neurotoxin gene cluster on a plasmid may be linked to TA system genes. TA systems were first recognized in prokaryotic plasmids as mechanisms to ensure stable inheritance (Ogura and Hiraga 1983). Typically, two adjacent plasmid genes encode a stable toxin, which persists in the cytoplasm, together with its labile antitoxin, which has to be expressed constitutively to avoid cell death (Buts et al. 2005). This ensures postsegregational killing of any daughter cell that fails to receive a plasmid. Because a plasmid-free daughter cell is a very unlikely event when high copy-number plasmids partition, TA systems are only found on low- to medium-copy plasmids, consistent with the estimate of 2–5 copies for neurotoxin-encoding plasmids of Group II *C. botulinum* type B4. The fact that three strains in the present study were shown to have lost their neurotoxin gene, presumably via plasmid loss, suggests that in Group II *C. botulinum* type B4 the plasmid maintenance system is not infallible. A significant outcome of this study is that the TA systems discovered on the type B4 plasmids may also be involved in preventing integration of the neurotoxin gene cluster into the bacterial chromosome. When located on the chromosome, TA systems often play a role in control of gene expression, bacterial population control and programmed cell death (Yamaguchi and Inouye 2009). Very subtle adjustments of the toxin: antitoxin ratio determines which of these outcomes occur (Yamaguchi et al. 2011). The present study suggests that Group II *C. botulinum* type B4 also possess a chromosomally located TA system; a homologue of a PemI–PemK module (Tsuchimoto et al. 1988; Agarwal et al. 2010). If the plasmid-borne TA genes are functional, there may be a fine balance between any toxin produced from chromosomal TA genes, and any antitoxin produced from the plasmid. Integration of the neurotoxin gene cluster-bearing plasmid into the chromosome would result in loss of selection for unintegrated plasmids, eventually decreasing the plasmid-associated TA gene copy number from potentially five to only one. This would affect any balance of the chromosomal: plasmid toxin: antitoxin ratio, with major consequences for the cell. The *pemI* gene of the PemI–PemK TA system is the only TA system gene shared by all 11 plasmids studied here. It is also located very close to the *ha* genes and so has a greater chance of cosegregation with the neurotoxin gene cluster during any plasmid recombination event. Together with the fact that a homologue of the *pemI-pemK* module is also carried by the chromosome adds strength to the hypothesis that this TA system may be involved in Group II *C. botulinum* type B4 plasmid maintenance. Because loss of the neurotoxin gene cluster plasmid can be observed, it may be that programmed cell death is not the sole outcome. It would be interesting to test this hypothesis by measuring parameters such as growth rate in these examples.

In summary, all strains of Group II *C. botulinum* type B studied to date possess a type B4 neurotoxin gene located in an *ha* cluster located on a plasmid, with no examples found on the chromosome. This strongly contrasts with Group I *C. botulinum* and Group II *C. botulinum* type E, where the botulinum neurotoxin gene may be located either on the chromosome or on a plasmid. Three classes of type B4 plasmid (47–63 kb) have been found, with one class apparently dominant in marine locations, and another class dominant in European terrestrial environments. It remains to be established whether possession of these plasmids can be linked with the ability to thrive in these different environments. A third class of plasmid is less common and is a hybrid between the other two other classes, suggesting that the opportunity for contact between the seemingly geographically separated populations does occur. Sequencing of many more examples of Group II *C. botulinum* type B4 will be required to address these questions. Interestingly, the type B4 plasmids or the neurotoxin clusters that they carry have not been transmitted to other clostridia or even to strains of Group II *C. botulinum* types E or F. A TA system may account for retention of the plasmid and also its inability to integrate into the chromosome. The present detailed study of the type B4 plasmids of Group II *C. botulinum* firmly links genetically based subdivisions to geographical origin. This information is not only of great importance for the study of the evolution of this human pathogen but might also prove vital for determination of the provenance of newly isolated strains.
